# Delayed Spontaneous Regression of Metastatic Gastric Cancer: A Case Report of a Rare Finding

**DOI:** 10.7759/cureus.20224

**Published:** 2021-12-07

**Authors:** Mansoor Zafar, Abdul Wahab Paracha, Muteeb Ashraf, Tila Muhammad, Mark Whitehead, Muhammad Toqeer

**Affiliations:** 1 Gastroenterology and Hepatology, Conquest Hospital. East Sussex Healthcare NHS Trust, St Leonards-on-Sea, GBR; 2 General Internal Medicine-Gastroenterology, Grange University Hospital, Cwmbran, GBR; 3 General Internal Medicine-Gastroenterology, Conquest Hospital. East Sussex Healthcare NHS Trust, St Leonards-on-Sea, GBR; 4 Gastroenterology, Conquest Hospital. East Sussex Healthcare NHS Trust, St Leonards-on-Sea, GBR

**Keywords:** esophagogastroduodenoscopy (egd), computed tomogram abdomen-pelvis, orogastroduodenoscopy, delayed spontaneous regression, metastatic stomach cancer, gastric adenocarcinoma

## Abstract

We discuss the case of a 74-year-old male who was referred with episodes of vomiting, nausea, and weight loss. Ultrasound abdomen had suggested multiple liver metastases and a triple-phase CT scan of abdomen-pelvis confirmed the same. His oesophago-gastro-duodenoscopy (OGD), also known as upper endoscopy (EGD), showed a large ulcerated lesion at the lesser curvature, which was later confirmed to be poorly differentiated adenocarcinoma on biopsy. The patient was started on palliative chemotherapy, which he tolerated poorly, but a CT scan had suggested a minimal reduction in the size of liver metastasis. He was given two cycles of chemotherapy; however, due to poor tolerance and unresponsiveness to chemotherapy, he was referred to palliative care.

The patient declined any medical support for the next six years, after which he visited his general practitioner (GP) for a follow-up review. Routine blood tests showed new-onset mild iron deficiency anaemia. He denied any symptoms. He was referred to Gastroenterology for repeat OGD, and it showed a tiny nodular area in the stomach at the site of previous cancer, which was reported as non-specific chronic inflammation on biopsy, and CT abdomen showed a marked reduction in size and number of liver metastases. On further clinical review, he reported feeling well and his anaemia resolved without any intervention.

## Introduction

Spontaneous regression of a malignant tumour is a very rare finding [1]. It has been estimated that this occurs in one in 100,000 people with cancer and about 20 such cases have been reported annually [2]. Spontaneous regressions of a malignant tumour have often been reported in hypernephroma, neuroblastoma, malignant melanoma, choriocarcinoma, bladder cancer, but it is extremely rare in stomach cancers [3,4]. In this report, we present a case of spontaneous regression of metastatic gastric cancer in an elderly male patient. We also engage in a review of the related literature.

## Case presentation

**Figure 1:**
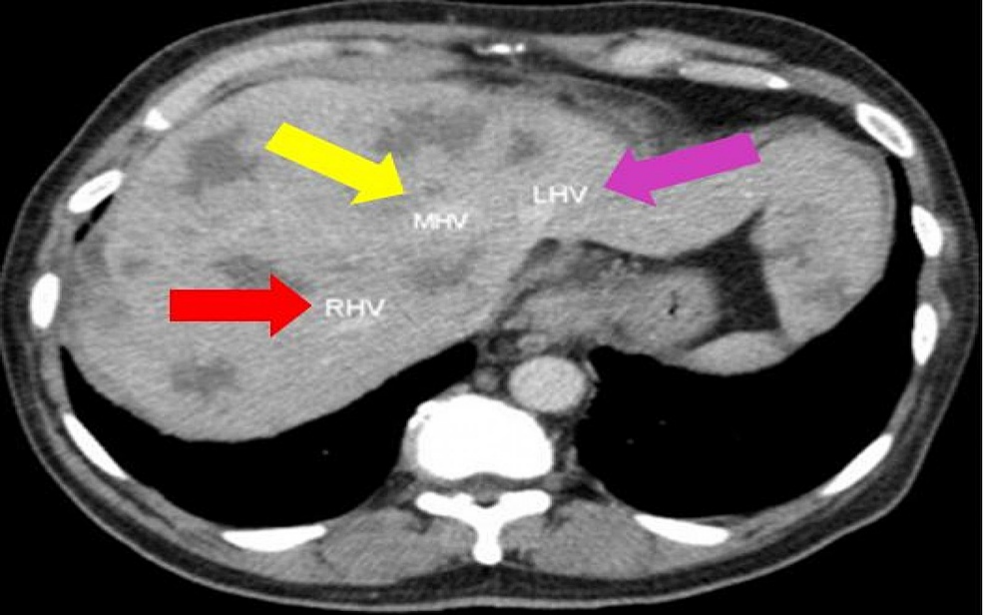
CT cross-sectional view of the abdomen shows widespread metastasis of stomach cancer to the liver and portal area. The arterial phase demonstrates an early enhancement CT: computed tomography; MHV: middle hepatic vein (yellow arrow); RHV: right hepatic vein (red arrow); LHV: left hepatic vein (pink arrow)

**Figure 2:**
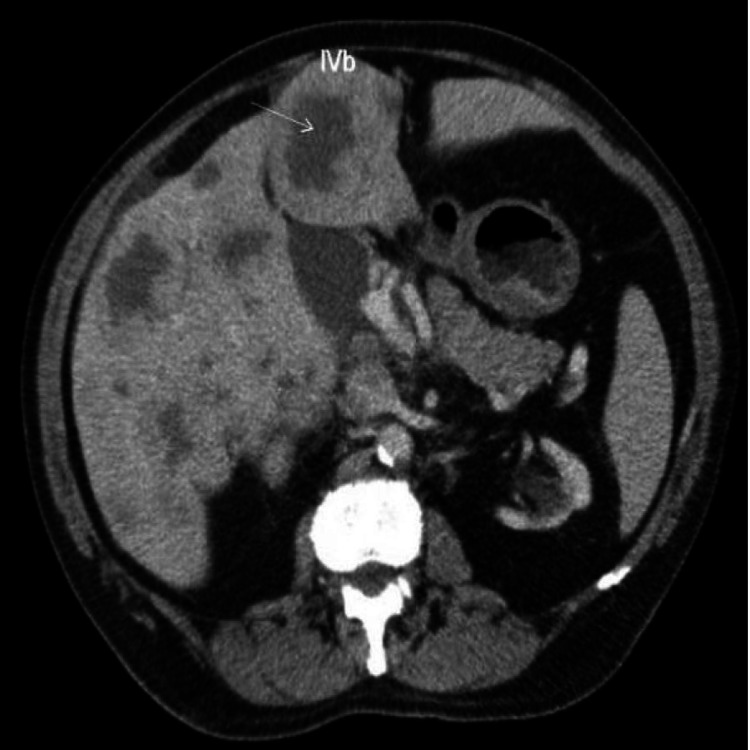
CT cross-sectional view of the abdomen shows widespread metastasis of stomach cancer to the liver and portal area. A delayed washout pattern of the mass is also seen (white arrow) CT: computed tomography; IV b: segment IV (inferior) lateral to the falciform ligament

A 74-year-old male of South Asian descent, with a history of nausea, vomiting, and unintentional weight loss, was referred by his general practitioner (GP) to the Gastroenterology outpatient department as an urgent case. The patient had a past medical history of diabetes mellitus type 2, chronic kidney disease (CKD), and hypertension, which were all well-controlled with medical therapy. He did not have any family history of gastrointestinal or any other malignancies. An ultrasound of the abdomen organized by his GP had suggested multiple liver metastases. He underwent a gastroscopy and CT scan to confirm the findings seen on the ultrasound. His CT scan confirmed multiple liver metastases (Figures 1, 2).

**Figure 3:**
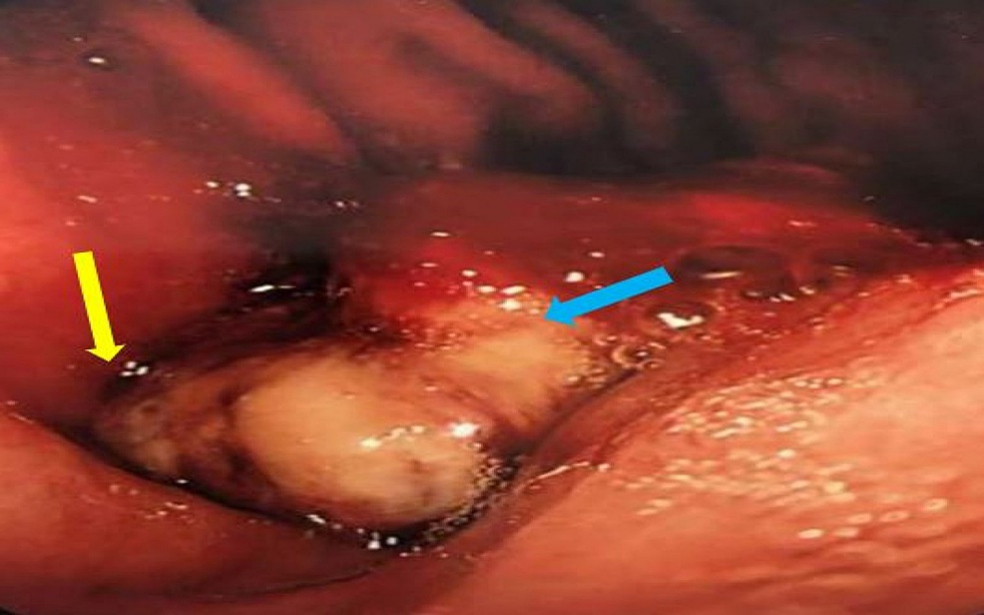
Oesophago-gastro-duodenoscopy showing fungating stomach cancer (blue arrow), and almost near-complete obstruction of stomach outlet (yellow arrow)

**Figure 4:**
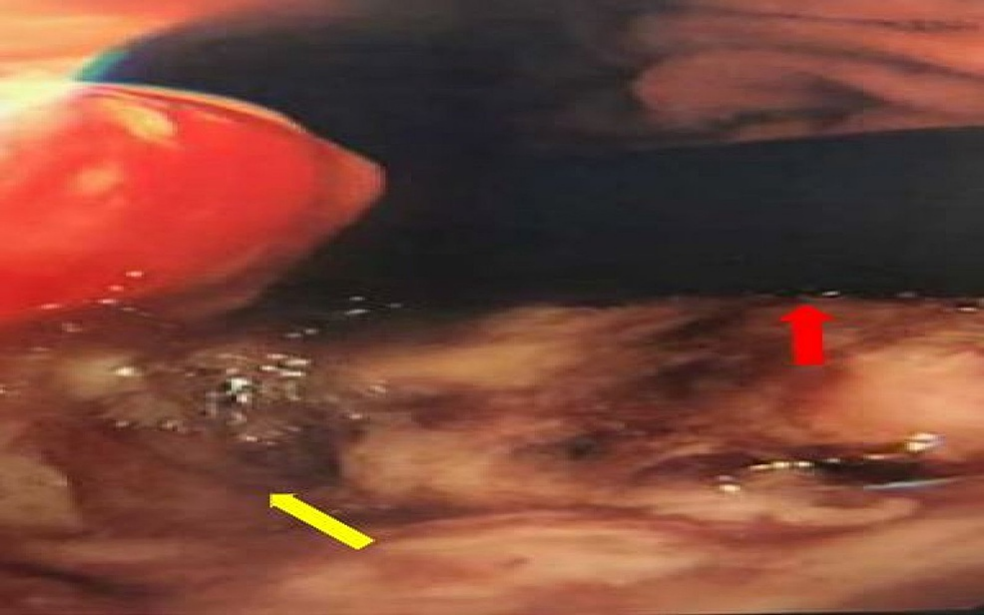
Oesophago-gastro-duodenoscopy with scope in retroflexion (red arrow), with fungating large stomach cancer (yellow arrow)

The gastroscopy showed large malignant-looking ulceration at the lesser curvature of the stomach (Figures 3, 4), which on biopsy was reported as poorly differentiated adenocarcinoma. His case was discussed in the multidisciplinary meeting (MDM) and a diagnosis of gastric adenocarcinoma (histopathology) with widespread metastasis (radiological) to the liver was made, and an oncology review was recommended. Following the oncology review, the patient was started on palliative chemotherapy with epirubicin, oxaliplatin, and capecitabine. He tolerated the first cycle of chemotherapy well but felt really unwell with nausea and vomiting with the second cycle, and hence it was stopped. On further review in the oncology clinic, it was agreed that further chemotherapy will not bring any benefit and the patient was referred to the community palliative team. He was subsequently not followed up in the oncology clinic.

**Figure 5:**
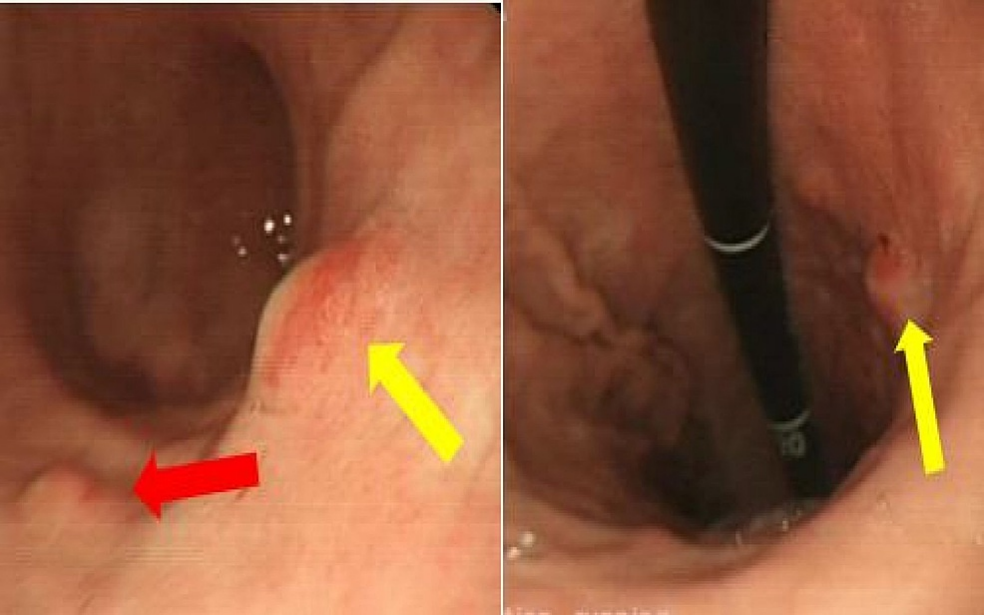
Oesophago-gastro-duodenoscopy direct (A) and in retroflexion (B) performed six years after the initial presentation The images show the complete resolution of stomach cancer. Only a few benign gastric polyps (red arrow) with mild erythematous gastritis (yellow arrow) are seen

The patient declined all medical support for the next six years. Later on, he reported to his GP for a follow-up and he was again referred by his GP to the Gastroenterology team to evaluate for mild iron deficiency anaemia. He denied any major symptoms apart from mild indigestion. His diabetes control was adequate. His full blood count showed a haemoglobin level of 119 g/L (normal range: 130-180 g/L), blood iron level of 10 mcmol/L (10.74-30.43), and ferritin of 10 µg/L (13-150). His blood reticulocyte count, vitamin B12, and folate levels were normal. His faecal immunochemistry test (FIT), also known as faecal occult blood test, was negative. His creatinine was slightly elevated at 130 mcmol/L (59-104), consistent with CKD. His liver function tests were completely normal. Given his background of metastatic gastric carcinoma, he agreed to be investigated again with a gastroscopy and CT scan of the chest, abdomen, and pelvis. The gastroscopy showed no signs of malignancy apart from a small nodular area at the lesser curvature of the stomach. Biopsies from this area were reported as showing chronic inflammation only. Since he presented with Iron deficiency anaemia with low haemoglobin levels, he further underwent colonoscopy, which was inconclusive. Repeat OGD and biopsies that were taken four weeks later showed similar changes (Figure 5).

**Figure 6:**
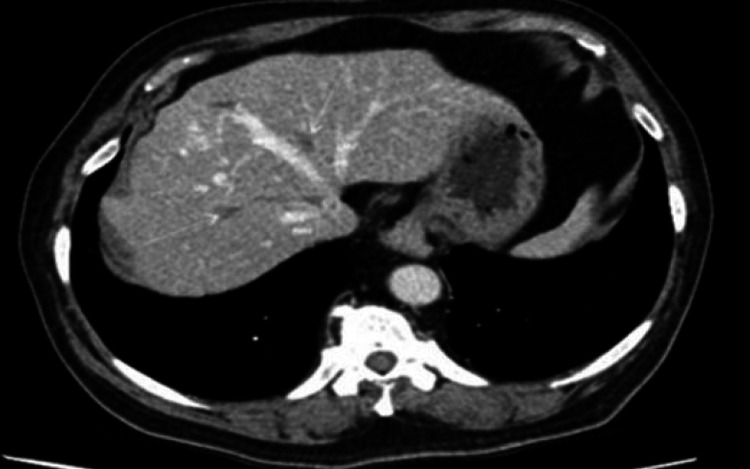
CT abdomen cross-sectional view The image shows complete resolution of stomach cancer with complete disappearance of metastasis along the liver and portal area six years after the initial presentation CT: computed tomography

**Figure 7:**
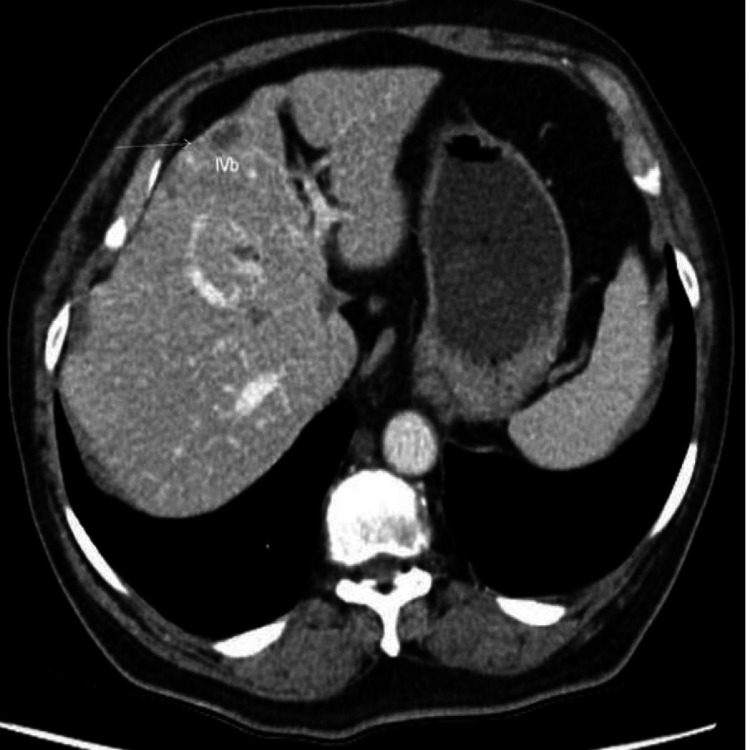
CT abdomen cross-sectional view triple-phase (triphase) The image shows complete resolution of stomach cancer with complete disappearance of metastasis along the liver and portal area six years after the initial presentation (white arrow) CT: computed tomography; IV b: segment IV (inferior) lateral to the falciform ligament

CT scan of the chest, abdomen, and pelvis showed a marked reduction in size and number of liver metastases when compared with CT scan six years earlier (Figures 6, 7). There was no evidence of metastatic disease elsewhere. His hospital identification was reconfirmed against his stomach biopsy samples. On further follow-up in the Gastroenterology clinic, he continued to feel well and his anaemia had resolved with a haemoglobin level of 140 g/L without any intervention. No further action was recommended apart from a routine follow-up at three months. The patient was well with no evidence of anaemia on the blood tests, and he was discharged from the Gastroenterology clinic at that time. He was seen again in the clinic two years later and found to be absolutely asymptomatic.

## Discussion

The estimated incidence of cancer regression is less than 1:100,000 cases [1]. A systematic review performed between the years 1900 and 1987 revealed that different types of tumours regress with varying frequencies. For instance, certain malignancies like hypernephroma, malignant melanoma, neuroblastoma, retinoblastoma, leukaemia, lymphoma, breast cancer, bladder cancer, and choriocarcinoma are found to have a higher incidence of spontaneous resolution [2,3]. Challis and Stam have established that spontaneous regression of gastric carcinomas is extremely rare [3]. Various pathophysiological mechanisms have been proposed to understand the process of regression, which include immune mediation, tumour inhibition by growth factors and/or cytokines, induction of differentiation, hormonal mediation, elimination of a carcinogen, tumour necrosis and/or angiogenesis inhibition, psychological factors, apoptosis, and epigenetic mechanisms [4,5]. The Everson and Cole study, which reviewed the literature on spontaneous regression of tumours, has described 167 malignancies with spontaneous regression of which four were gastric cancers [6].

It is well-established that almost all tumour types and metastases can regress spontaneously although certain histological types regress more than others. Spontaneous regression of cancer was originally defined by Everson and Cole as "the partial or complete disappearance of a malignant tumour in the absence of all treatment or in the presence of therapy which is considered to be inadequate to exert a significant influence on neoplastic disease" [1,6-8].

Gastric carcinoma is one of the most common types of cancer in the world. Based on GLOBOCAN 2018 data, stomach cancer is the fifth most common neoplasm, with an incidence of 5.7%, and the third most deadly cancer [9]. Our review of the literature revealed that approximately 20 cases of spontaneous gastric cancer regression have been reported so far [4].

Regression of metastatic stomach cancer has been reported post-surgery or after undergoing chemotherapy from 10 days to up to six months. We presented a rare case of advanced metastatic gastric carcinoma that spontaneously regressed six years after the diagnosis with advanced metastatic stage. The regression occurred without any significant intervention apart from one cycle of chemotherapy. Although an argument could be made that the patient may have already had regression prior to his presentation after six years, our report covers the period when he sought medical attention again after six years of his initial presentation. Hence, we do not have any way to prove that an earlier regression had occurred. Another argument could be made with respect to the possibility of the patient having other conditions besides cancer, with IgG4 disease possibly the only candidate in this regard from a radiological perspective. However, the biopsy findings from the stomach along with normal biopsy findings on repeat endoscopy rule out the possibility of IgG4 disease. Additionally, biopsy samples were double-analysed both times, and the patient does not have a biological twin, The patient has not been reported to have any recurrence of symptoms eight years later. To conclude, we could not identify any significant reason for the resolution of his cancer.

## Conclusions

Spontaneous regression of cancers is a rare occurrence but is still considered a successful outcome. The regression of cancers is frequently underreported: our case, for instance, was reported a few years after the confirmed regression. The follow-up of patients remains an important factor in terms of continuous learning and motivation toward achieving optimal outcomes for both the patients and the clinicians.
